# Circulating CCR7+ICOS+ Memory T Follicular Helper Cells in Patients with Multiple Sclerosis

**DOI:** 10.1371/journal.pone.0134523

**Published:** 2015-07-31

**Authors:** Xueli Fan, Tao Jin, Songchen Zhao, Caiyun Liu, Jinming Han, Xinmei Jiang, Yanfang Jiang

**Affiliations:** 1 Neuroscience Center, Department of Neurology, the First Hospital of Jilin University, Jilin University, Changchun, China; 2 Genetic Diagnosis Center, the First Hospital of Jilin University, Jilin University, Changchun, China; 3 Key Laboratory for Zoonosis Research, Ministry of Education, the First Hospital of Jilin University, Jilin University, Changchun, China; 4 Jiangsu Co-innovation Center for Prevention and Control of Important Animal Infectious Diseases and Zoonosis, Yangzhou, China; INSERM, FRANCE

## Abstract

**Objective:**

This study is aimed at examining the potential roles of circulating memory T follicular helper (Tfh) cells in patients with multiple sclerosis (MS).

**Methods:**

The numbers of different subsets of circulating memory Tfh cells in 25 patients with relapsed MS before and after treatment as well as 14 healthy controls (HC) were examined by flow cytometry. The levels of plasma IL-21 in all patients and cerebrospinal fluid (CSF) IL-21 in some MS patients and controls with non-inflammatory neuronal diseases (NND) were measured by ELISA.

**Results:**

In comparison with that in the HC, the numbers of circulating CD3+CD4+CXCR5+CD45RA-, ICOS+, CCR7+ and CCR7+ICOS+ memory Tfh cells and the levels of plasma IL-21 significantly increased in MS patients, but significantly decreased in the patients with complete remission (CR). The levels of CSF IL-21 were significantly higher in the MS patients than that in the NND patients. The numbers of CCR7+ICOS+ memory Tfh cells were positively correlated with the EDSS scores, the levels of plasma and CSF IL-21, IgG, MBP-Ab or MOG-Ab.

**Conclusions:**

Our findings indicated that circulating memory Tfh cells, especially CCR7+ICOS+ memory Tfh cells, may be associated with the relapse of MS and may serve as a new therapeutic target.

## Introduction

Immune memory is the hallmark of acquired immune response. Memory T cells are of great importance to rapidly mediate a potent secondary immune response to antigens and participate in the pathogenesis of recurrent autoimmune diseases, hypersensitivity, and vaccination [1]. There are two subsets of memory T cells. While effector memory (CCR7- memory) T cells that migrate to inflamed peripheral tissues and provide a rapid immune response, and central memory (CCR7+ memory) T cells have little effector function, predominantly home to secondary lymphoid organs, proliferate and differentiate into effector cells [2].

Multiple sclerosis (MS) is a relapse and remission autoimmune disease in the central nervous system (CNS), leading to damage to the myelin and axons of the brain and spinal cord [3]. The etiology and mechanisms underlying the development and relapse of MS are poorly understood. Although MS is thought to be a T cell-mediated autoimmune disease [4,5] and both CD4+ and CD8+ T cells are crucial for the pathogenesis of MS, humoral immunity is indispensable in the development of MS [3,6–8]. Indeed, B and plasma cells infiltrate and meningeal B-cell follicles are present in the CNS [3,6,9,10], and autoantibodies against myelin basic protein (MBP) and myelin oligodendrocyte glycoprotein (MOG) exist in MS patients [6,11,12]. However, how T cell immunity regulates humoral responses during the pathogenesis of MS has not been clarified.

T follicular helper (Tfh) cells are a subgroup of CD4+ T cells and are important to regulate humoral immunity. The functional development of Tfh cells is regulated by transcription factor B cell lymphoma 6 (Bcl-6). Functionally, Tfh cells can promote the germinal center (GC) formation, B cell differentiation and antibody production [13].Tfh cells express chemokine receptor CXC-chemokine receptor 5 (CXCR5), CXCR3, CCR6, CCR7, programmed death-1 (PD-1), CD40 ligand (CD40L), inducible costimulator (ICOS), SAP (signaling lymphocytic activation molecule associated protein), and secrete interleukin 21 (IL-21) [14–16]. Tfh cells can become memory CD45RA-CD4+CXCR5+ Tfh cells, which can be subdivided into different subsets, dependent on CCR6, CXCR3, PD-1, CCR7 and ICOS expression. Previous studies have shown that aberrant activation of Tfh responses is associated with the development of systemic lupus erythematosus [17,18], rheumatoid arthritis [19] and MS [20]. However, the role of memory Tfh cells in the relapse of MS has not been clarified. Indeed, it is unclear whether the numbers of different subsets of circulating memory Tfh cells are changed in MS patients and how the change in the numbers of different subsets of circulating Tfh cells is associated with the relapse of MS in humans. Furthermore, it is unclear how the numbers of different subsets of circulating memory Tfh cells are associated with the severity of MS and the levels of IL-21, and autoantibodies, particularly in the cerebrospinal fluid (CSF) of MS patients. Methylprednisolone has been regularly used for treatment of relapse of MS in the clinic [21]. However, it is unknown how the methylprednisolone treatment modulates the numbers of different subsets of circulating Tfh cells.

In this study, we examined the numbers of different subsets of circulating memory Tfh cells in MS patients before and after treatment with methylprednisolone and the levels of plasma and CSF IL-21 and autoantibodies. In addition, we explored the potential relationships among the values of these measures to unravel the potential role of different subsets of memory Tfh cells in the relapse of MS. We found significantly increased numbers of CCR7+ICOS+ memory Tfh cells and increased levels of plasma and CSF IL-21 in patients with relapsed MS. The numbers of circulating CCR7+ICOS+ memory Tfh cells were correlated positively with the EDSS scores, the levels of plasma and CSF IL-21, MBP-Ab or MOG-Ab in MS patients. Treatment with methylprednisolone significantly decreased the numbers of CCR7+ICOS+ memory Tfh cells and the levels of plasma IL-21 in patients with complete remission (CR) of MS. Our data indicated that CCR7+ICOS+ memory Tfh cells participated in the relapse of MS and may serve as new therapeutic targets for intervention of MS.

## Materials and Methods

### Patients and controls

A total of 25 patients with relapsed MS were recruited in the inpatient service of the Department of Neurology, the First Hospital of Jilin University (Changchun, China) during July 2014 to March 2015. These patients were diagnosed, according to the 2010 McDonald’s diagnostic criteria [22]. All patients had new neurological symptoms and signs lasting at least 24 hours as well as new lesions examined on MRI. Individual patients were excluded if she/he had another autoimmune disease, recent infection or received corticosteroid or immunosuppressant therapy during the past 4 weeks. The disease severity of individual patients was evaluated by the Expanded Disability Status Scale (EDSS). Fourteen age- and gender-matched healthy controls (HC) were recruited in the Physical Examination Center of the hospital. Their demographic and clinical characteristics are shown in Table 1. Among these MS patients, there were 15 patients who received a lumbar puncture. In addition, another 10 gender and age-matched patients with non-inflammatory neurological diseases (NND) were recruited and received a lumbar puncture as the controls. Demographic and clinical features of MS and NND patients who received a lumbar puncture are shown in Table 2. Written informed consent was obtained from individual participants. This study was approved by Medical Ethics Committee of the First Hospital of Jilin University, Changchun, China (No.2014-244).

**Table 1:** The demographic and clinical features of MS patients and HC.

	MS	HC
n	25	14
Age (year)	40 (23–59)	38.5 (18–56)
Female/male	17/8	9/5
ARR	1 (0.2–2)	
Duration of disease (year)	2 (1–30)	
No of attack	2 (1–6)	
EDSS scores	2 (0.5–4.5)	
WBC (10^9^/L)	6.79 (4.24–9.44)	6.6 (4.9–8.3)
Lymphocytes (10^9^/L)	1.83 (1.14–2.72)	1.56 (1.25–2.91)

Data shown are median and range, except specified. ARR: Annual relapse rate; EDSS: Expanded disability status scale; MS: Multiple sclerosis; WBC: White blood cells. Normal values: WBC, 3.5–9.5 ×10^9^/L; Lymphocytes, 1.1–3.2 ×10^9^/L.

**Table 2:** The demographic and clinical features of MS patients and NND who received a lumbar puncture.

	MS	NND
n	15	10
Age (year)	36 (23–56)	37 (27–50)
Female/male	13/2	9/1
		7—headache
Diagnosis	MS	3—seizure
CSF WBC (10^6^/L)	11 (1–37) *	1.5 (0–6)
CSF proteins (g/L)	0.36 (0.28–0.65) *	0.29 (0.16–0.41)
CSF IgG (mg/l)	47.9 (17.1–149) *	22 (10–32)
Plasma MBP-Ab (OD value)	0.152 (0.062–3.288)	
CSF MBP-Ab (OD value)	0.11 (0.055–0.381)	
Plasma MOG-Ab (OD value)	0.167 (0.065–3.363)	
CSF MOG-Ab (OD value)	0.081 (0.056–0.308)	

Data shown are median and range, except specified. CSF: Cerebrospinal fluid; MBP-Ab: Antibodies against myelin basic protein; MOG-Ab: Antibodies against myelin oligodendrocyte glycopotein; NND: Non-inflammatory neurological diseases; OD: Optical density; WBC: White blood cell. Normal values: CSF WBC, 0–8 ×10^6^/L; CSF proteins, 0.15–0.45 g/L; CSF IgG, 0–34 mg/l; Plasma MBP-Ab < 0.75; CSF MBP-Ab < 0.65; Plasma MOG-Ab < 0.64; CSF MOG-Ab < 0.56. *P<0.05 vs. the HC.

### Blood and cerebrospinal fluid sampling and analyses

Fasting venous blood samples were collected from individual healthy controls and MS patients before and 4–8 weeks after treatment. One portion of blood was used for preparing peripheral blood mononuclear cells (PBMCs) by density-gradient centrifugation using Lymphoprep (Axis-Shield PoC AS, Oslo, Norway), and the remaining blood samples were centrifuged for preparing plasma samples. The cerebrospinal fluid (CSF) samples were collected from 15 MS patients and 10 NND when they underwent a lumbar puncture. The numbers of white blood cell (WBC) and lymphocytes in peripheral blood were routine examined and the numbers of WBC in the CSF, and the concentrations of proteins and IgG in CSF were tested. In addition, the levels of plasma and CSF antibodies against MBP (MBP-Ab) or against MOG (MOG-Ab) in individual MS patients were measured by enzyme-linked immunosorbent assay (ELISA).

### Treatment and follow up

Twenty MS patients were first treated intravenously with methylprednisolone (1000 mg/day for three days, 500 mg/day on day 4–6, 250 mg/day on day 7–9; 120 mg/day on day 10–12) and then treated orally with 60 mg/day methylprednisolone for 3 days, followed by gradually decreased doses (60 mg/day on day 13–15; 30mg/day on day 16–18; 15mg/day on day 19–21 and gradually decreased doses). The patients visited the outpatient office 4–8 weeks after treatment and their blood samples were collected for further examinations. A total of 12 patients were back for the follow up and their clinical characteristics are shown in Table 3.

**Table 3:** The demographic and clinical features of 12 MS patients after treatment.

	MS-CR	MS-PR
n	7	5
Age (year)	27 (23–40)	42 (25–50)
Female/male	7/0	4/1
CSF WBC (10^6^/L)	11 (1–11)	12 (4–37)
CSF proteins (g/L)	0.31 (0.28–0.45)	0.4 (0.32–0.65)
CSF IgG (mg/l)	37.8 (22.5–72.3)	84.7 (38.6–149) *

Data shown are median and range, except specified. CR: Complete remission; CSF: Cerebrospinal fluid; PR: Partial remission; WBC: White blood cells. Normal values: CSF WBC, 0–8 ×10^6^/L; CSF proteins, 0.15–0.45 g/L; CSF IgG, 0–34 mg/l. *P<0.05 vs. the HC.

### Flow cytometry analysis (FCM)

Human PBMCs at 10^6^/tube were stained in duplicate with APC-H7-anti-CD3, BV510-anti-CD4, FITC-anti-CD45RA, PE-Cy7-anti-CCR7, PerCP-Cy5.5-anti-CXCR5, PE-anti-ICOS, BV421-anti-PD-1, PE-CF594-anti-CD154, or isotype-matched control IgG (Becton Dickinson, San Diego, USA) at room temperature for 30 minutes, respectively. After being washed with PBS, the cells were subjected to flow cytometry analysis in a BD FACSAria Ⅱ (BD Biosciences, San Jose, CA USA) and data were analyzed with FlowJo software (v7.6.2). At least 50,000 events per sample were analyzed. The cells were gated initially on living lymphocytes, on CD3+ and CD4+, and then on CXCR5+ and CD45RA-. Subsequently, the frequency of ICOS+, PD-1+ or CD40L+ CD3+CD4+CXCR5+CD45RA- T (memory Tfh) cells were analyzed. In addition, the CD3+CD4+CXCR5+CD45RA- Tfh cells were gated on CCR7 and the frequency of ICOS+, PD-1+, CD40L+ CCR7+ or CCR7- Tfh cells was analyzed. The numbers of each type of memory Tfh cells in individual subjects were calculated by the counts of lymphocytes per liter of blood multiplied by the percentage of different subsets of memory Tfh cells in lymphocytes.

### Measurement of plasma and CSF IL-21 by ELISA

The levels of plasma and CSF IL-21 were measured by ELISA using a specific kit according to the manufacture’s instructions (Multi Sciences Biotech Co., Hangzhou, China). The detection limit for human IL-21 was 11.99 pg/ml.

### Statistical Analysis

Data are expressed as median and range. The difference between HC and MS patients was analyzed by Fisher exact test and Kruskal Wallis test, and the difference between MS patients before and after treatment was analyzed by Wilcoxon test using the SPSS 19.0 software. The relationship between variables was evaluated by the Spearman rank correlation test. A two-side *P* value of <0.05 was considered statistically significant.

## Results

### The numbers of circulating memory Tfh cells in MS patients and HC

**Fig 1:**
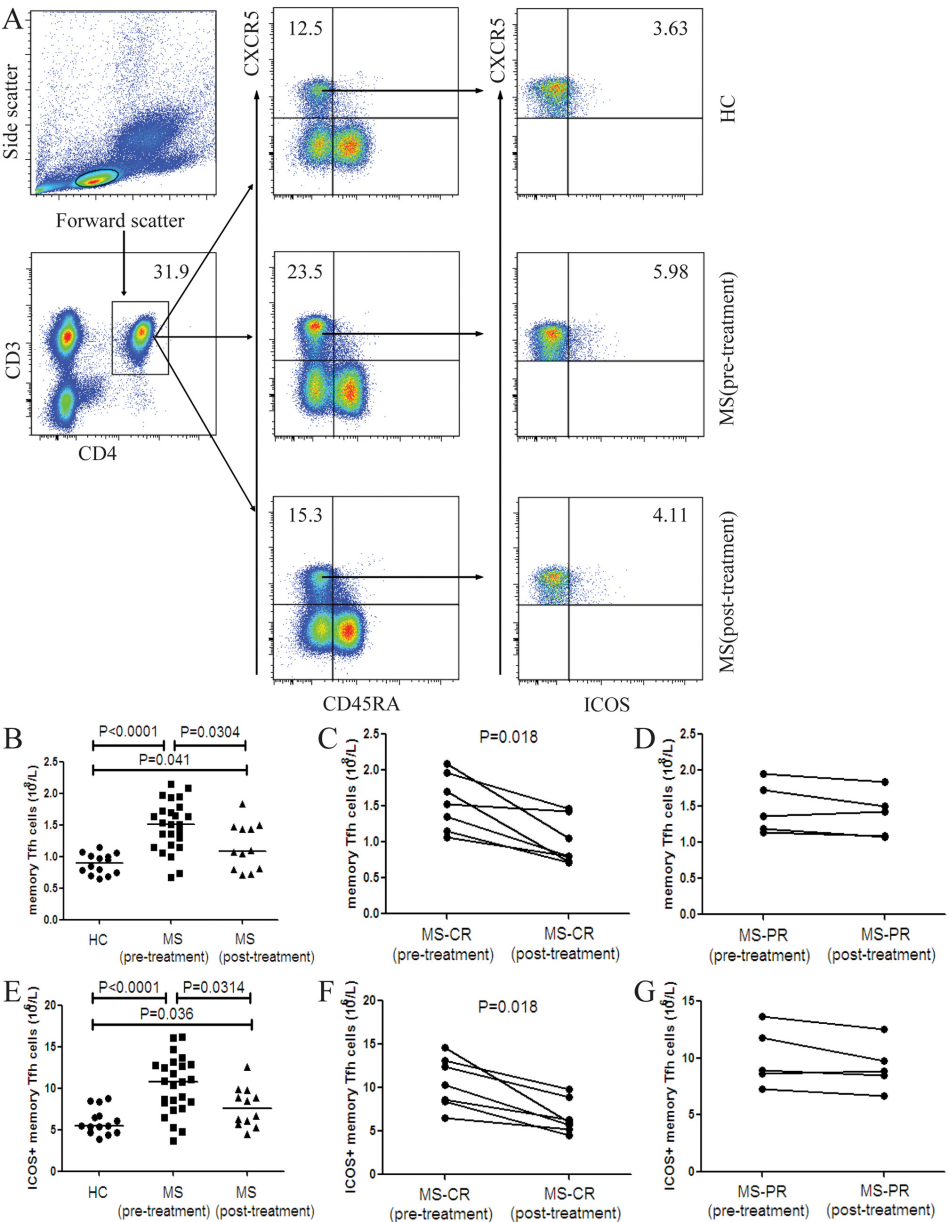
FACS analysis of the numbers of circulating memory Tfh cells and ICOS+ memory Tfh cells in individual subjects. PBMCs were isolated from individual subjects and stained with different fluorescent antibodies. The cells were gated sequentially on living lymphocytes, CD3+ and CD4+, and then on CXCR5+ and CD45RA- cells. The frequency of CD3+CD4+CXCR5+CD45RA- (memory) Tfh cells was determined. Subsequently, memory Tfh cells were gated on ICOS and the frequency of memory Tfh and ICOS+ memory Tfh cells in lymphocytes was analyzed and the numbers of each type of cells in total lymphocytes per liter were calculated. (A) Flow cytometry analysis. (B) The numbers of memory Tfh cells in the HC, MS patients pre- and post-treatment. (C) The numbers of memory Tfh cells in the MS-CR patients pre- and post-treatment. (D) The numbers of memory Tfh cells in the MS-PR patients pre- and post-treatment. (E) The numbers of ICOS+ memory Tfh cells in the HC, MS patients pre- and post-treatment. (F) The numbers of ICOS+ memory Tfh cells in the MS-CR patients pre- and post-treatment. (G) The numbers of ICOS+ memory Tfh cells in the MS-PR patients pre- and post-treatment. There was no significant difference in the numbers of PD-1+, PD-1+ICOS+ and CD40L+ memory Tfh cells between the patients and HC as well as in MS patients pre- and post-treatment (data not shown). The horizontal lines indicate the median values for each group.

We analyzed the numbers of circulating different subsets of memory Tfh cells (CD3+CD4+CXCR5+CD45RA- T cells) in MS patients before and after treatment as well as in HC by flow cytometry. The numbers of memory Tfh cells and ICOS+ memory Tfh cells in MS patients before and after treatment were significantly greater than that in the HC although the numbers of these cells in MS patients after treatment were significantly reduced (Fig 1A, 1B and 1E). Among 12 patients with post-treatment follow-up, seven patients achieved complete remission and five patients did partial remission (PR). We found that the numbers of memory Tfh cells and ICOS+ memory Tfh cells in patients who achieved CR significantly decreased, as compared with that before treatment (Fig 1C and 1F). However, such decreased trend was not found in patients with PR post-treatment compared with that pre-treatment (Fig 1D and 1G). In addition, there was no significant difference in the numbers of PD-1+, PD-1+ICOS+ and CD40L+ memory Tfh cells between the patients and HC as well as in MS patients before and after treatment (Data not shown).

### The numbers of circulating CCR7+ memory Tfh cells in MS patients and HC

**Fig 2:**
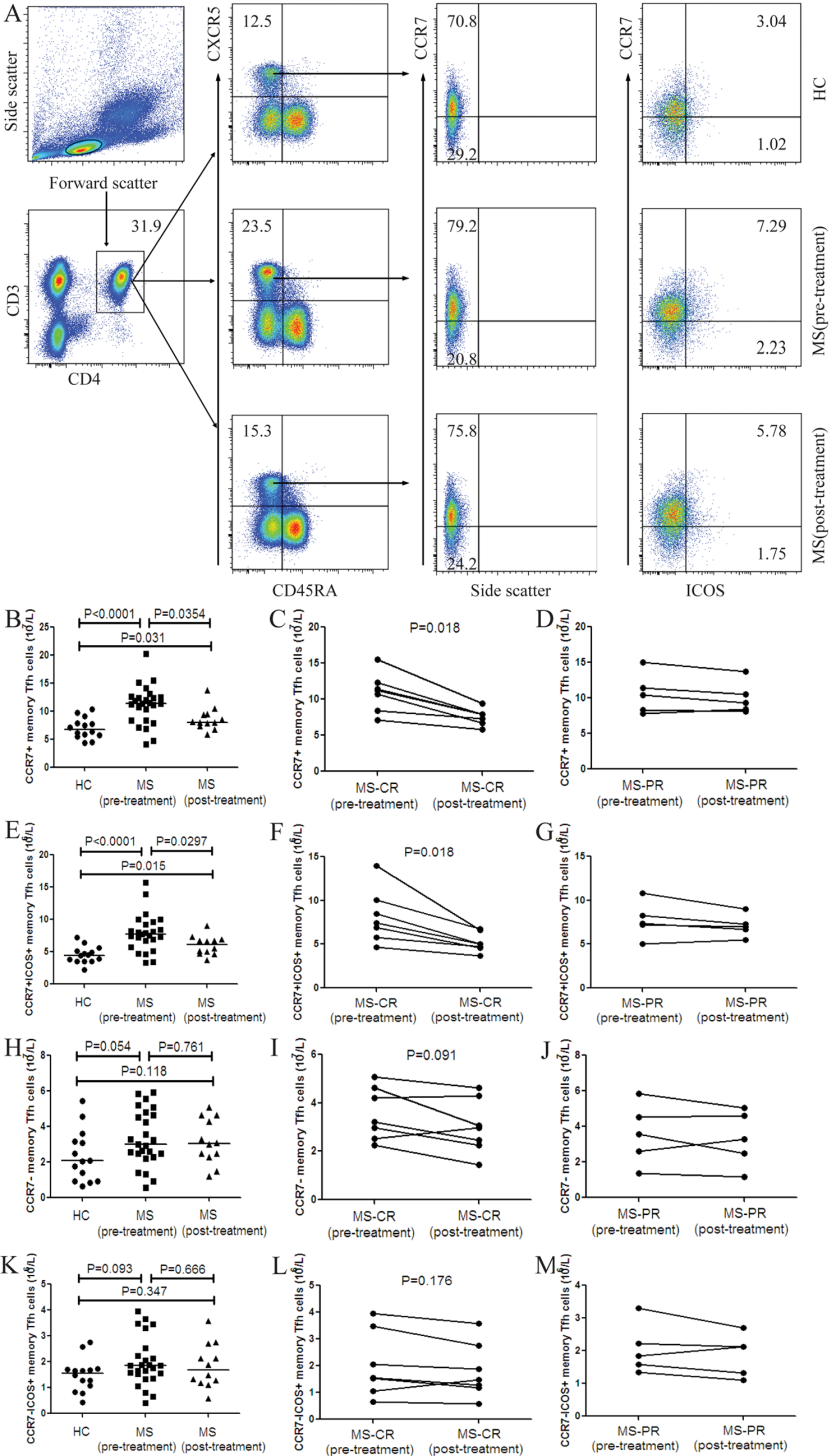
FACS analysis of the numbers of circulating CCR7+ and CCR7+ICOS+ memory Tfh cells in individual subjects. After being stained with different fluorescent antibodies, the CD3+CD4+CXCR5+CD45RA- T cells were gated. Subsequently, memory Tfh cells were gated on CCR7 expression and the frequency of CCR7+, CCR7+ICOS+, CCR7- and CCR7-ICOS+ memory Tfh cells was analyzed by flow cytometry and the numbers of each type of cells were calculated. (A) Flow cytometry analysis. (B) The numbers of CCR7+ memory Tfh cells in the HC, MS patients pre- and post-treatment. (C) The numbers of CCR7+ memory Tfh cells in the MS-CR patients pre- and post- treatment. (D) The numbers of CCR7+ memory Tfh cells in the MS-PR patients pre- and post-treatment. (E) The numbers of CCR7+ICOS+ memory Tfh cells in the HC, MS patients pre- and post-treatment. (F) The numbers of CCR7+ICOS+ memory Tfh cells in the MS-CR patients pre- and post-treatment. (G) The numbers of CCR7+ICOS+ memory Tfh cells in the MS-PR patients pre- and post-treatment. (H) The numbers of CCR7- memory Tfh cells in the HC, MS patients pre- and post-treatment. (I) The numbers of CCR7- memory Tfh cells in the MS-CR patients pre- and post- treatment. (J) The numbers of CCR7- memory Tfh cells in the MS-PR patients pre- and post-treatment. (K)The numbers of CCR7-ICOS+ memory Tfh cells in the HC, MS patients pre- and post-treatment. (L) The numbers of CCR7-ICOS+ memory Tfh cells in the MS-CR patients pre- and post-treatment. (M) The numbers of CCR7-ICOS+ memory Tfh cells in the MS-PR patients pre- and post-treatment. There was no significant difference in the numbers of CCR7-PD-1+, CCR7-PD-1+ICOS+, CCR7-CD40L+, CCR7+PD-1+, CCR7+PD-1+ICOS+, CCR7+CD40L+ memory Tfh cells between the patients and HC as well as in MS patients before and after treatment (data not shown). The horizontal lines indicate the median values for each group.

Next, the numbers of circulating different subsets of CCR7+ and CCR7- memory Tfh cells in individual subjects were determined by flow cytometry analysis. We found that the numbers of CCR7+ and CCR7+ICOS+ memory Tfh cells in MS patients after treatment were significantly lower than that in those patients before treatment, but remained significantly higher than that in the HC (Fig 2A, 2B and 2E). Furthermore, the numbers of CCR7+ and CCR7+ICOS+ memory Tfh cells in the MS patients with CR outcome were significantly lower than that before treatment (Fig 2C and 2F). However, such decreased trend was not found in patients with PR outcome after treatment (Fig 2D and 2G). Furthermore, there was no significant change in the numbers of CCR7-, CCR7-ICOS+ memory Tfh cells between the MS patients before and after treatment or between the patients and HC (Fig 2H and 2K). Moreover, there was no decreased trend in the numbers of CCR7- and CCR7-ICOS+ memory Tfh cells in the MS patients with CR or PR after treatment (Fig 2I, 2J, 2L and 2M). In addition, there was no significant difference in the numbers of CCR7-PD-1+, CCR7-PD-1+ICOS+, CCR7-CD40L+, CCR7+PD-1+, CCR7+PD-1+ICOS+, CCR7+CD40L+ memory Tfh cells between the patients and HC as well as in MS patients before and after treatment (Data not shown).

### The levels of plasma and CSF IL-21 in MS patients

**Fig 3:**
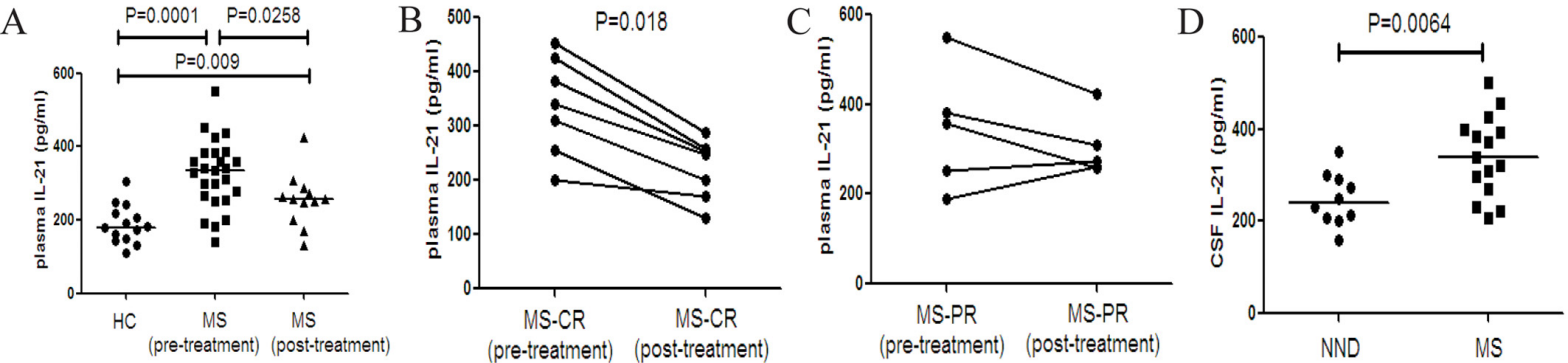
Analysis of plasma and CSF IL-21 in MS patients before and after treatment. The levels of plasma and CSF IL-21 in individual subjects were tested by ELISA. (A) The levels of plasma IL-21 in the HC, MS patients pre- and post-treatment. (B) The levels of plasma IL-21 in the MS-CR patients pre- and post- treatment. (C) The levels of plasma IL-21 in the MS-PR patients pre- and post- treatment. (D) The levels of CSF IL-21 in the MS patients pre-treatment and NND patients. Data are expressed as the mean values of individual samples. The horizontal lines represented the median values.

The levels of plasma IL-21 in all the subjects, and the levels of CSF IL-21 in 15 MS patients and 10 patients with NND were measured by ELISA. We found that the levels of plasma IL-21 were higher in the MS patients before treatment than that in the MS patients after treatment and that in the HC (Fig 3A). The levels of plasma IL-21 in the MS patients with CR after treatment significantly decreased, as compared with that before treatment (Fig 3B). However, such decreased trend was not detected in patients with PR after treatment (Fig 3C). In addition, the levels of CSF IL-21 were significantly higher in the MS patients than in the NND patients (Fig 3D).

### The correlations between the numbers of different subsets of circulating memory Tfh cells and the values of clinical measures in MS patients

**Fig 4:**
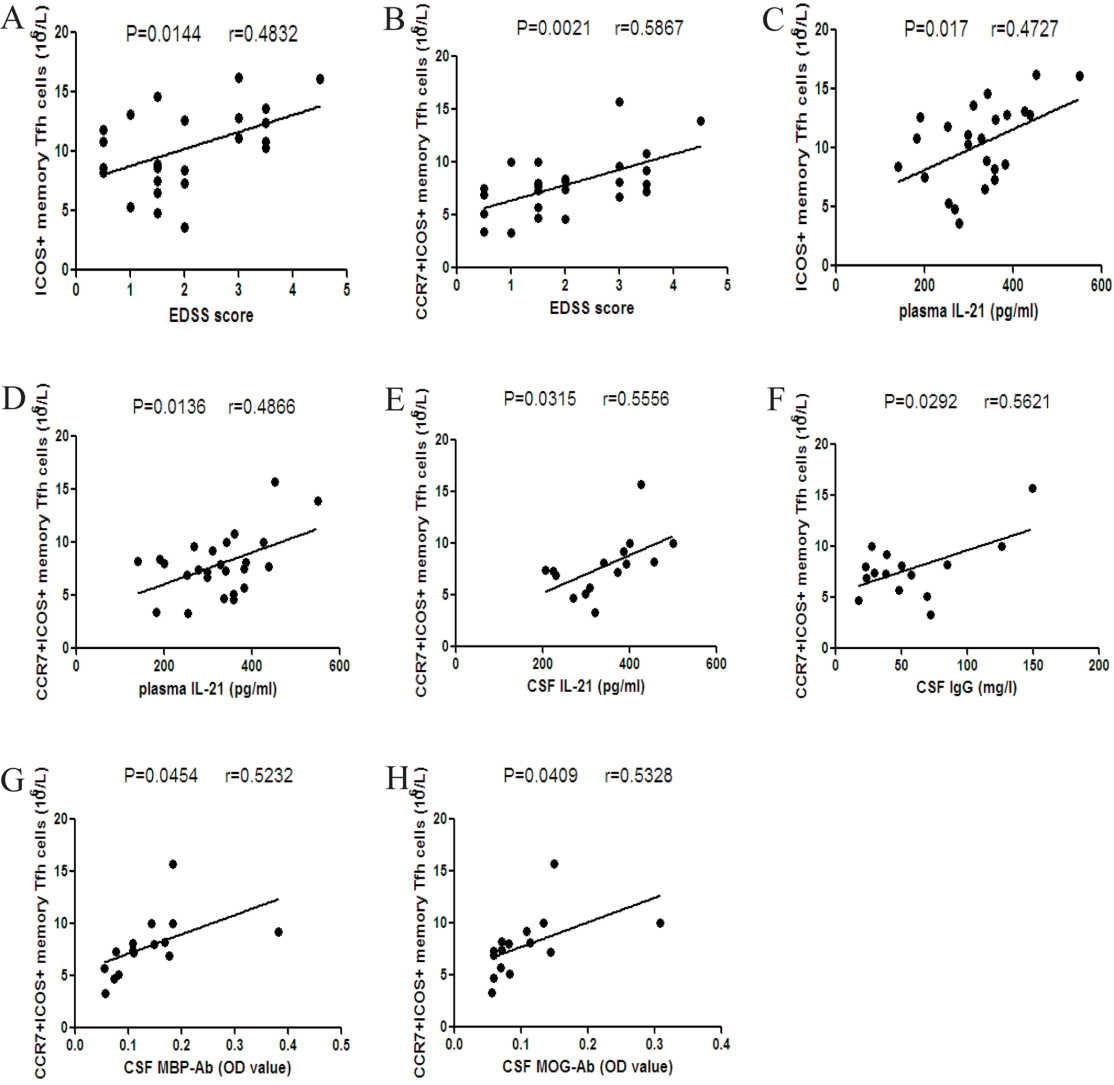
Correlation analysis of the numbers of different subsets of circulating memory Tfh cells and the values of clinical measures in MS patients. (A-B) The numbers of ICOS+ and CCR7+ICOS+ memory Tfh cells were positively associated with the EDSS scores in MS patients. (C-D) The numbers of ICOS+ and CCR7+ICOS+ memory Tfh cells were positively associated with the levels of plasma IL-21. (E-F) The numbers of CCR7+ICOS+ memory Tfh cells were positively correlated with the levels of CSF IL-21 and CSF IgG. (G-H) The numbers of CCR7+ICOS+ memory Tfh cells were positively correlated with the levels of CSF MBP-Ab and CSF MOG-Ab.

To understand the potential importance of memory Tfh cells, we analyzed the relationship between the numbers of memory Tfh cells and the values of clinical measures tested in the MS patients. We found that the numbers of ICOS+ and CCR7+ICOS+ memory Tfh cells were positively correlated with the EDSS scores and the levels of plasma IL-21 in the MS patients (Fig 4A–4D). Furthermore, there was a significant correlation between the number of CCR7+ICOS+ memory Tfh cells and the levels of CSF IL-21 or IgG in the MS patients (Fig 4E and 4F). In addition, the numbers of CCR7+ICOS+ memory Tfh cells were positively correlated with the levels of CSF MBP-Ab and CSF MOG-Ab (Fig 4G and 4H). However, there was no correlation among the values of other measures tested.

## Discussion

In our study, we found that there were significantly greater numbers of circulating memory Tfh cells, ICOS+, CCR7+ and CCR7+ICOS+ memory Tfh cells, accompanied by higher levels of plasma IL-21 in MS patients, as compared with that in the HC. Similarly, the levels of CSF IL-21 were significantly higher in the MS patients than in the NND patients. The numbers of ICOS+ and CCR7+ICOS+ memory Tfh cells were positively correlated with the EDSS scores and the levels of plasma IL-21 in the MS patients. The numbers of CCR7+ICOS+ memory Tfh cells were positively associated with the levels of CSF IL-21, IgG, MBP-Ab and MOG-Ab in MS patients. Treatment with corticosteroids significantly decreased the numbers of different subsets of memory Tfh cells and the levels of plasma IL-21 in MS patients with CR outcome. These results indicated that circulating memory Tfh cells, especially CCR7+ICOS+ memory Tfh cells may participate in the relapse of MS and be used for evaluating the severity of MS relapse. Obviously, CCR7+ICOS+ memory Tfh cells may contribute to immune responses during the pathogenesis of MS relapse in the CNS.

Following antigen stimulation, the majority of activated CD4+ T cells die via apoptosis, while some activated CD4+ T cells survive and become memory T cells [1]. When faced with antigen re-challenge, effector memory (CCR7- memory) T cells migrate to inflamed peripheral tissues and provide a rapid and effective immune response, whereas CCR7+ memory T cells migrate to secondary lymphoid organs where they proliferate and differentiate into effector cells [2]. Memory Tfh cells can circulate in vivo and participate in the process of autoimmunity by promoting humoral responses [15,23,24]. We found that significantly increased numbers of ICOS+ memory Tfh cells in the MS patients, consistent with a previous report [20]. ICOS+ activated Tfh cells can interact with ICOSL on B cells, and promote the formation of GC and the differentiation of B cells as well as antibody production [25–27]. Contrary to our results, another study showed there was no significant difference in the frequency of circulating Tfh cells in CD4+ T cells between MS and HC [28]. The difference may be because the patients we studied were different from those they examined [28]. While we recruited only relapsed MS patients they studied MS patients at relapse and remission. Hence, ICOS+ memory Tfh cells may be important memory Tfh cells, associated with the relapse of MS.

IL-21, as the major cytokine produced by Tfh cells, plays a major role in Tfh cell survival, and B cell proliferation, survival and differentiation in the GC [29]. A previous study has shown higher levels of plasma IL-21 in MS patients [28] and the levels of IL-21 expression by PBMCs are reduced in MS patients after mitoxantrone treatment [20]. Furthermore, the polymorphism of IL-21R is associated with the development of MS [30] and higher levels of IL-21 expression are detected in CD4+ T cell infiltrates in acute active brain lesions of MS patients [31]. We also detected significantly higher levels of plasma and CSF IL-21 in MS patients. Our findings support the notion that IL-21 may participate in the relapse process of MS in the CNS.

Among circulating memory Tfh cells, CCR7+ memory Tfh cells can migrate into B cell follicles and promote humoral responses [32], and activated CCR7-PD-1+ memory Tfh cells in secondary lymphoid tissues also enhance humoral responses, associated with the development of autoimmune diseases [33]. Intriguingly, our results indicated significantly increased numbers of CCR7+ and CCR7+ICOS+, but not CCR7-PD-1+, memory Tfh cells in MS patients and the numbers of CCR7+ICOS+ memory Tfh cells were positively correlated with the EDSS scores, the levels of plasma and CSF IL-21, the levels of CSF MBP-Ab and MOG-Ab in MS patients. CCR7+ and CCR7+ICOS+ memory Tfh cells can migrate into secondary lymphoid organs [14,15,24]. CCR7 is a chemokine receptor that is known to regulate lymphocyte migration [34,35] and CCR7 is vital for the trafficking of lymphocytes participating in the CNS immunosurveillance [36,37]. A previous study has revealed that CCR7+CD4+ memory T cells exist in the CSF of MS patients [38]. Another study has shown that meningeal B-cell follicles are related to severe pathological changes, rapid disease progression, and poor prognosis in MS patients [10]. Accordingly, we speculate that CCR7+ICOS+ memory Tfh cells may migrate into the CNS, where they promote the development of ectopic B-cell follicles and antibody production. Hence, CCR7+ICOS+ memory Tfh cells may be crucial for the pathological progression in the CNS of MS patients.

Corticosteroids can inhibit inflammation via the down-regulation of T and B cell function. Recently, a study has demonstrated that corticosteroids promote Tfh cell apoptosis by regulating IL-21 and IL-6 through plasma glucocorticoid-regulated kinase 1 (SGK 1) in SLE [18]. We found that treatment with corticosteroid significantly decreased the numbers of memory Tfh cells, ICOS+, CCR7+ and CCR7+ICOS+ memory Tfh cells and the levels of plasma IL-21 in MS patients with CR. Our results further indicated that memory Tfh cells were involved in the pathogenic progression of MS. Further studies are needed to illuminate the molecular mechanisms underlying the action of corticosteroid in regulating Tfh cells in MS patients.

In conclusion, our data suggest that circulating memory Tfh cells may participate in the pathogenic progression of MS. Furthermore, CCR7+ICOS+ memory Tfh cells may play a crucial role in the autoimmune inflammation lesions in the CNS and may serve as a new biomarker for evaluating the disease activity and severity in patients with relapsed MS. Hence, CCR7+ICOS+ memory Tfh cells may be new therapeutic targets for treatment of MS. We recognized that our studies had limitations, including a small sample size, the lack of long-term follow-up, no functional studies of different subsets and little information from affected tissues in the CNS. Therefore, further researches on the roles of CCR7+ICOS+ memory Tfh cells in the pathogenesis of MS in a bigger population are of the essence.
